# Clinical aspects of cervical cancer patients treated in 2010 in “Saint Spiridon” University Hospital from Iasi, Romania

**DOI:** 10.1186/1753-6561-6-S4-P20

**Published:** 2012-07-09

**Authors:** SO Salceanu, A Murariu, O Murariu, DPT Iancu

**Affiliations:** 1 University of Medicine and Pharmacy ”Grigore T. Popa”, Iasi, Romania; 2 “Saint Spiridon” University Hospital, Iasi, Romania

## Introduction

Romania occupies the first place in Europe concerning cervical cancer incidence and mortality (European Cancer Observatory - 2008 cancer fact sheet). Our objective is to give a preliminary assessment of the cervical cancer situation in the Eastern part of Romania.

## Methods

In 2010 we collected data from 94 new patients with cervical cancer (age 28 - 85) who were treated in “Saint Spiridon” University Hospital from Iasi, Romania. 41 of the patients were from several cities from Romania (Barlad, Botosani, Iasi, Pascani, Radauti, Roman, Suceava, Vaslui) and 53 patients were from rural areas.

## Results

The staging was done according to the FIGO classification: 14,89% of the patients were stage I, 46,80% stage II, 26,59% stage III and 11,70% of the patients were stage IV. The biopsy results showed that 85,10% of all patients had squamous cell carcinoma, 9,57% adenocarcinoma and 5,31% adenosquamous carcinoma. Multiparity was 86,17% among the cervical cancer patients and only 21,27% of the patients were smokers. 20,21% of the patients had a family history of malignancies. Blood tests were performed and 57,44% of the patients had secondary anaemia and 26,59% had leucopenia. The debut of the cervical cancer consisted in: abnormal bleeding in 85,10% of patients, pelvic pain in 47,87% of patients and 28,72% of women had purulent vaginal discharge. Other debut symptoms include: dyspareunia, abdominal cramps, nausea, frequent urination, etc. 67,03% of patients were treated using surgery, chemotherapy and external beam radiation and in 32,97% of women palliative chemotherapy and external beam radiation were used.

## Conclusions

The early detection of the malignant disease is the most efficient and effective strategy to successfully fight cervical cancer. This preliminary study based only on patients treated in 2010 shows that the screening programme must be improved. It is important to include a bigger number of patients so that we can draw a conclusion based on statistical significant data. It is also important to develop quality-of-life and cervical cancer risk factors questionnaires in order to asses better the cervical cancer situation in Romania.

